# Crystal structure and Hirshfeld surface analysis of (C_7_H_9_N_4_O_2_)[ZnCl_3_(H_2_O)]

**DOI:** 10.1107/S2056989020002753

**Published:** 2020-03-10

**Authors:** Hicham El Hamdani, Mohamed El Amane, Mohamed Saadi, Lahcen El Ammari

**Affiliations:** a Equipe Metallation, Complexes Moleculaires et Applications, Université Moulay Ismail, Faculté des Sciences, BP 11201 Zitoune, 50000 Meknés, Morocco; b Laboratoire de Chimie Appliquée des Matériaux, Centre des Sciences des Matériaux, Faculty of Sciences, Mohammed V University in Rabat, Avenue Ibn Batouta, BP 1014, Rabat, Morocco

**Keywords:** crystal structure, mol­ecular salt, hydrogen bonding

## Abstract

In the title mol­ecular salt, 1,3-dimethyl-2,6-dioxo-2,3,6,7-tetra­hydro-1*H*-purin-9-ium aqua­tri­chlorido­zincate(II), (C_7_H_9_N_4_O_2_)[ZnCl_3_(H_2_O)], the fused ring system of the cation is close to planar, with the largest deviation from the mean plane being 0.037 (3) Å. In the complex anion, the Zn^II^ cation is coordinated by three chloride ions and one oxygen atom from the water ligand in a distorted tetra­hedral geometry. In the crystal, inversion dimers between pairs of cations linked by pairwise N—H⋯O hydrogen bonds generate *R*_2_^2^(10) rings. The anions are linked into dimers by pairs of O—H⋯Cl hydrogen bonds and the respective dimers are linked by O—H⋯O and N—H⋯Cl hydrogen bonds. Together, these generate a three-dimensional supra­molecular network. Hirshfeld surfaces were generated to gain further insight into the packing.

## Abstract 2

In the title mol­ecular salt, (C_7_H_9_N_4_O_2_)[ZnCl_3_(H_2_O)], the crystal packing exhibits O—H⋯O, O—H⋯Cl, N—H⋯O and N—H⋯Cl hydrogen bonds.

## Chemical context   

Theophylline, C_7_H_8_N_4_O_2_, is an alkaloid derivative of xanthine, containing a fused pyrimidine-imidazole ring system with conjugated double bonds. It has many biological and pharmacological properties (see, for example, Rao *et al.*, 2005 ▸; Piosik *et al.*, 2005 ▸). Various studies have shown that theophylline can be used as a medicine for the treatment of asthmatic bronchitis and chronic obstructive bronchitis (under several brand names), and as anti­cancer drugs (Nafisi *et al.* 2003 ▸; Rao *et al.* 2005 ▸; Piosik *et al.* 2005 ▸). Furthermore, theophylline complexes with transition metals can be used in anti­cancer drugs (David *et al.*, 1999 ▸).
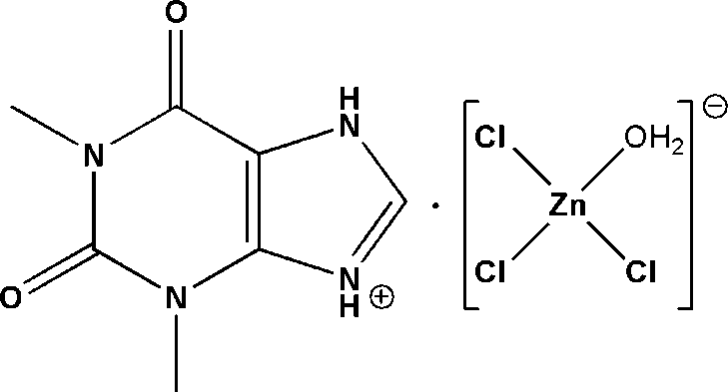


As part of our studies in this area, we reacted theophylline with ZnCl_2_ under acid conditions to give the mol­ecular salt (C_7_H_9_N_4_O_2_)·[ZnCl_3_(H_2_O)] and its crystal structure is described herein.

## Structural commentary   

**Figure 1:**
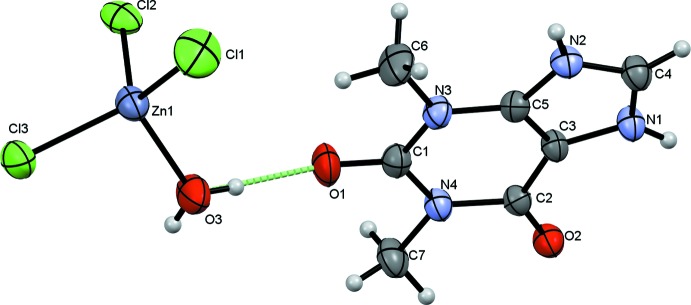
Mol­ecular structure of the title compound with displacement ellipsoids drawn at the 50% probability level.

The asymmetric unit of the title mol­ecular salt (Fig. 1 ▸) comprises one theophyllinium (C_7_H_9_N_4_O_2_)^+^ cation protonated at N2 and one [ZnCl_3_(H_2_O)]^−1^ anion. As expected, the [ZnCl_3_(H_2_O)] tetra­hedron contains one short Zn—O bond distance [2.0240 (15) Å] and three longer Zn—Cl bonds distances [in the range 2.2121 (7)–2.2745 (6) Å]. These bond lengths are consistent with the values observed in analogous compounds such as [H_3_N(CH_2_)_8_NH_3_]ZnCl_4_, [C_6_H_5_–C_2_H_4_–NH_3_]_2_ZnCl_4_, (C1_2_H_12_N_2_)[ZnCl_4_] and (C_10_H_22_N_2_)[ZnCl_4_](El Mrabet *et al.*, 2017 ▸), as are the Cl—Zn—Cl [111.45 (3)–116.99 (3)°] and Cl—Zn—O [101.36 (5)–108.19 (5)°] bond angles (Kassou *et al.*, 2016 ▸; Campos-Gaxiola *et al.*, 2015 ▸; Soudani *et al.*, 2013 ▸).

## Supra­molecular features   

**Figure 2:**
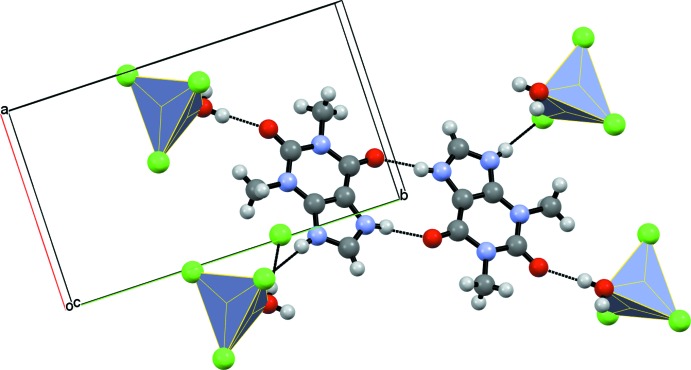
Mol­ecules linked by O–H⋯O and O–H⋯Cl strong hydrogen bonds.

**Figure 3:**
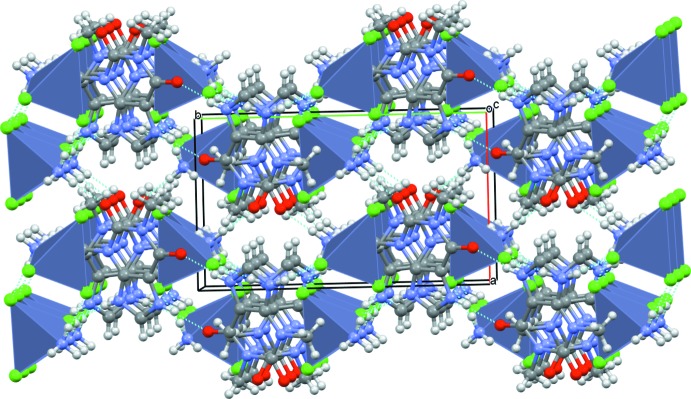
Perspective view of the crystal structure along the *c* axis showing the layered organization.

The packing is consolidated by a network of hydrogen bonds (Table 1 ▸, Fig. 2 ▸). The cations are linked into inversion dimers by pairs of N1—H1⋯O2 hydrogen bonds, which generate 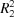
(10) rings. The anions also form inversion dimers, being linked by pairwise O3—H3*A*⋯Cl3 hydrogen bonds. The anions are linked to the cations *via* O3—H3*B*⋯O1 hydrogen bonds from the water mol­ecule to a carbonyl group of the pyrimidine ring. Finally, the cations are linked to the anions *via* N2—H2⋯Cl2 hydrogen bonds. Taken together, these hydrogen bonds generate a three-dimensional supra­molecular network (Fig. 3 ▸), which also features short Cl⋯π contacts [Cl⋯centroid distances in the range of 3.533 (2)–3.620 (2) Å].

## Database survey   

A search of the Cambridge Structural Database (CSD, Version 5.40, May 2019; Groom *et al.*, 2016 ▸) for organic–inorganic compounds containing theophilinium in the cation revealed three similar structures: theophyllinium tri­chloro­theophyllineplatinum(II), bis­(theophyllinium) tetra­chloro­platinum(II) (Griffith *et al.*, 1979 ▸) and bis­(theophyllinium) tetra­bromo­palladium(II) (Salas *et al.*, 1989 ▸). In each of the three complexes, the metal cation is surrounded by four ligands in a planar geometry. The crystal structures of these compounds are different from that of the title compound; however, the organic–inorganic moities are linked through hydrogen bonds in all of these structures.

## Hirshfeld surface analysis   

In order to gain further insight into the inter­molecular inter­actions in the title compound, we used the program *Crystal Explorer* (Spackman & Jayatilaka, 2009 ▸), to consider separately the (C_7_H_9_N_4_O_2_)^+^ organic cation and the [ZnCl_3_(H_2_O)]^−^ inorganic anion.

**Figure 4:**
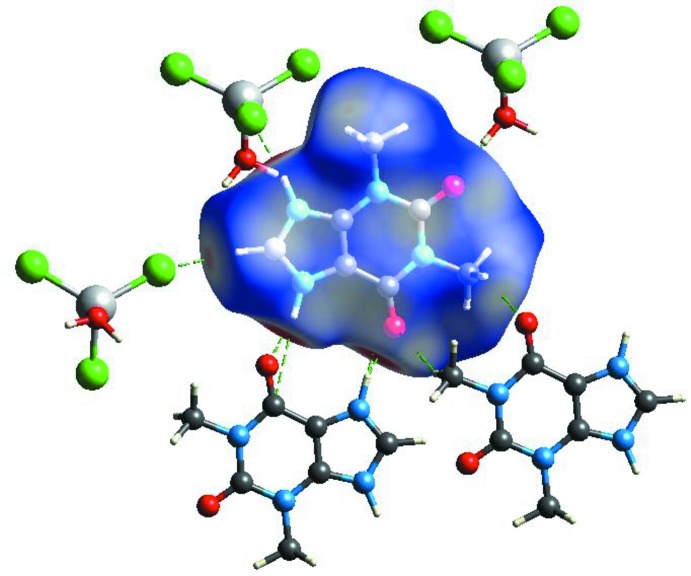
Hirshfeld d_norm_ surface of the (C_7_H_9_N_4_O_2_)^+^ cation in the title compound.

The Hirshfeld *d*_norm_ surface of the cation is depicted in Fig. 4 ▸. The most significant inter­actions are H⋯H (29.6%) contacts and the second largest percentage (25.8%) can be attributed to H⋯O inter­actions, which are responsible for the appearance of deep-red spots and correlate with the O—H⋯O and N—H⋯O hydrogen bonds. H⋯Cl (21.9%), C⋯Cl (8.1%), N⋯Cl (5.5%) and C⋯H (3.6%) inter­actions are also observed, with other contact types making a negligible contribution.

**Figure 5:**
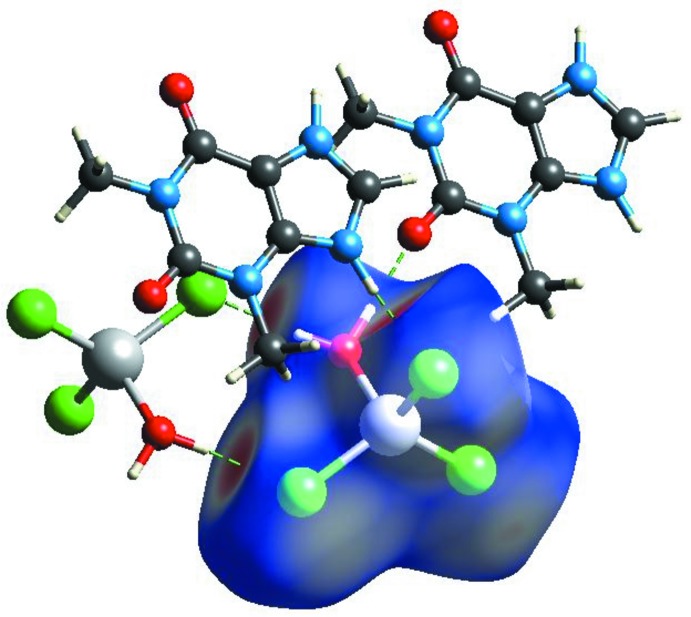
Hirshfeld *d*_norm_ surface of the [ZnCl_3_(H_2_O)]^−^ anion in the title compound.

The Hirshfeld surface of the [ZnCl_3_(H_2_O)] anion is depicted in Fig. 5 ▸ and shows red spots that correspond to the strong N—H⋯Cl and O—H⋯Cl hydrogen bonds: Cl⋯H contacts are the most abundant contributor to the surface at 54.7%. Other significant contributions include Cl⋯C (10.3%), H⋯H (9.6%), Cl⋯N (7.3%), H⋯O (5.6%) and H⋯Cl (4.7%). It is notable that the Cl⋯Cl contact percentage is 0%, *i.e*. the chloride anions avoid each other in the crystal.

## Synthesis and crystallization   

ZnCl_2_·6H_2_O (0.244 g, 1 mmol) was dissolved in 5 ml of water. Then, theophylline [C_7_H_8_N_4_O_2_] (0.180 g, 1 mmol) was dissolved in 3 ml of ethanol/water (1:1 *v*:*v*) with a few drops of conc. HCl (37%). The two solutions were mixed and after two weeks, colourless crystals of the title mol­ecular salt were obtained.

## Refinement   

Crystal data, data collection and structure refinement details are summarized in Table 2 ▸. The H atoms were all located in a difference map, but those attached to C and N atoms were repositioned geometrically (C—H = 0.93–0.96, N—H = 0.86 Å). The water H atoms were located in a difference map and refined as riding atoms in their as-found relative positions. The constraint *U*_iso_(H) = 1.2*U*_eq_(carrier) or 1.5*U*_eq_(C-meth­yl) was applied in all cases.

## Supplementary Material

Crystal structure: contains datablock(s) I. DOI: 10.1107/S2056989020002753/hb7891sup1.cif

Structure factors: contains datablock(s) I. DOI: 10.1107/S2056989020002753/hb7891Isup2.hkl

CCDC reference: 1986654

Additional supporting information:  crystallographic information; 3D view; checkCIF report

## Figures and Tables

**Table 1:** Hydrogen-bond geometry (Å, °)

*D*—H⋯*A*	*D*—H	H⋯*A*	*D*⋯*A*	*D*—H⋯*A*
N1—H1⋯O2^i^	0.86	1.87	2.7067 (18)	163
N2—H2⋯Cl2^ii^	0.86	2.21	3.0652 (15)	174
C6—H6*C*⋯O2^iii^	0.96	2.65	3.431 (3)	139
O3—H3*A*⋯Cl3^iv^	0.77	2.43	3.1915 (17)	176
O3—H3*B*⋯O1	0.82	1.90	2.718 (2)	173

Symmetry codes: (i) 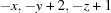; (ii) 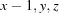; (iii) 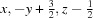; (iv) 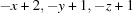.

**Table 2:** Experimental details

Crystal data	
Chemical formula	(C_7_H_9_N_4_O_2_)[ZnCl_3_(H_2_O)]
*M*_r_	370.92
Crystal system, space group	Monoclinic, *P*2_1_/*c*
Temperature (K)	296
*a*, *b*, *c* (Å)	8.0932 (14), 13.744 (3), 12.429 (2)
β (°)	92.290 (6)
*V* (Å^3^)	1381.4 (4)
*Z*	4
Radiation type	Mo *K*α
μ (mm^−1^)	2.36
Crystal size (mm)	0.32 × 0.25 × 0.11

Data collection	
Diffractometer	Bruker D8 VENTURE Super DUO
Absorption correction	Multi-scan (*SADABS*; Krause *et al.*, 2015 ▸)
*T*_min_, *T*_max_	0.587, 0.746
No. of measured, independent and observed [*I* > 2σ(*I*)] reflections	29522, 3046, 2753
*R*_int_	0.035

Refinement	
*R*[*F* ^2^ > 2σ(*F* ^2^)], *wR*(*F* ^2^), *S*	0.025, 0.070, 1.04
No. of reflections	3046
No. of parameters	166
H-atom treatment	H-atom parameters constrained
Δρ_max_, Δρ_min_ (e Å^−3^)	0.42, −0.26

Computer programs: *APEX3* and *SAINT* (Bruker, 2016 ▸), *SHELXT2014/5* (Sheldrick, 2015*a* ▸), *SHELXL2018/3* (Sheldrick 2015*b* ▸), *Mercury* (Macrae *et al.*, 2020 ▸) and *publCIF* (Westrip, 2010 ▸).

## supplementary crystallographic information

### Crystal data

**Table:**

(C_7_H_9_N_4_O_2_)[ZnCl_3_(H_2_O)]	*F*(000) = 744
*M**_r_* = 370.92	*D*_x_ = 1.783 Mg m^−^^3^
Monoclinic, *P*2_1_/*c*	Mo *K*α radiation, λ = 0.71073 Å
*a* = 8.0932 (14) Å	Cell parameters from 3046 reflections
*b* = 13.744 (3) Å	θ = 2.2–27.1°
*c* = 12.429 (2) Å	µ = 2.36 mm^−^^1^
β = 92.290 (6)°	*T* = 296 K
*V* = 1381.4 (4) Å^3^	Plate, colorless
*Z* = 4	0.32 × 0.25 × 0.11 mm

### Data collection

**Table:**

Bruker D8 VENTURE Super DUO diffractometer	3046 independent reflections
Radiation source: INCOATEC IµS micro-focus source	2753 reflections with *I* > 2σ(*I*)
HELIOS mirror optics monochromator	*R*_int_ = 0.035
Detector resolution: 10.4167 pixels mm^-1^	θ_max_ = 27.1°, θ_min_ = 2.2°
φ and ω scans	*h* = −10→10
Absorption correction: multi-scan (SADABS; Krause *et al.*, 2015)	*k* = −17→17
*T*_min_ = 0.587, *T*_max_ = 0.746	*l* = −15→15
29522 measured reflections	

### Refinement

**Table:**

Refinement on *F*^2^	Hydrogen site location: mixed
Least-squares matrix: full	H-atom parameters constrained
*R*[*F*^2^ > 2σ(*F*^2^)] = 0.025	*w* = 1/[σ^2^(*F*_o_^2^) + (0.0361*P*)^2^ + 0.7104*P*] where *P* = (*F*_o_^2^ + 2*F*_c_^2^)/3
*wR*(*F*^2^) = 0.070	(Δ/σ)_max_ = 0.001
*S* = 1.04	Δρ_max_ = 0.42 e Å^−^^3^
3046 reflections	Δρ_min_ = −0.26 e Å^−^^3^
166 parameters	Extinction correction: *SHELXL2018/3* (Sheldrick 2015b), Fc^*^=kFc[1+0.001xFc^2^λ^3^/sin(2θ)]^-1/4^
0 restraints	Extinction coefficient: 0.0116 (9)
Primary atom site location: dual	

### Special details

**Table:**

Geometry. All esds (except the esd in the dihedral angle between two l.s. planes) are estimated using the full covariance matrix. The cell esds are taken into account individually in the estimation of esds in distances, angles and torsion angles; correlations between esds in cell parameters are only used when they are defined by crystal symmetry. An approximate (isotropic) treatment of cell esds is used for estimating esds involving l.s. planes.

### Fractional atomic coordinates and isotropic or equivalent isotropic displacement parameters (Å^2^)

**Table:**

	*x*	*y*	*z*	*U*_iso_*/*U*_eq_	
C1	0.3890 (2)	0.73981 (12)	0.43314 (14)	0.0328 (4)	
C2	0.23025 (19)	0.88256 (11)	0.49620 (13)	0.0270 (3)	
C3	0.09587 (19)	0.84313 (11)	0.43243 (13)	0.0275 (3)	
C4	−0.1427 (2)	0.81114 (13)	0.35170 (15)	0.0347 (4)	
H4	−0.252141	0.816209	0.326593	0.042*	
C5	0.10920 (19)	0.75700 (12)	0.37968 (13)	0.0279 (3)	
C6	0.2625 (3)	0.61496 (16)	0.3140 (2)	0.0534 (6)	
H6A	0.175032	0.571204	0.331328	0.080*	
H6B	0.367334	0.584272	0.329585	0.080*	
H6C	0.253138	0.631212	0.238896	0.080*	
C7	0.5201 (2)	0.85870 (15)	0.55359 (17)	0.0427 (4)	
H7A	0.593033	0.889908	0.505244	0.064*	
H7B	0.575456	0.804184	0.587203	0.064*	
H7C	0.489054	0.904175	0.607817	0.064*	
N1	−0.06417 (17)	0.87524 (10)	0.41341 (12)	0.0314 (3)	
H1	−0.105499	0.928117	0.437743	0.038*	
N2	−0.04038 (17)	0.73720 (10)	0.33052 (12)	0.0317 (3)	
H2	−0.064969	0.686459	0.292687	0.038*	
N3	0.25046 (17)	0.70379 (10)	0.37848 (12)	0.0316 (3)	
N4	0.37091 (16)	0.82468 (10)	0.49296 (12)	0.0307 (3)	
O1	0.52249 (16)	0.69911 (11)	0.42937 (12)	0.0463 (3)	
O2	0.22824 (15)	0.95718 (8)	0.54976 (10)	0.0348 (3)	
O3	0.7194 (2)	0.54071 (11)	0.46116 (13)	0.0567 (4)	
H3A	0.791444	0.560052	0.496924	0.085*	
H3B	0.653875	0.586278	0.454444	0.085*	
Cl1	0.53785 (8)	0.37606 (5)	0.28558 (6)	0.06943 (19)	
Cl2	0.84322 (7)	0.55879 (3)	0.20036 (4)	0.04325 (13)	
Cl3	0.98173 (7)	0.36721 (4)	0.39782 (4)	0.05047 (15)	
Zn1	0.76669 (3)	0.45595 (2)	0.33282 (2)	0.03798 (10)	

### Atomic displacement parameters (Å^2^)

**Table:**

	*U*^11^	*U*^22^	*U*^33^	*U*^12^	*U*^13^	*U*^23^
C1	0.0309 (8)	0.0313 (8)	0.0364 (9)	0.0047 (7)	0.0020 (7)	0.0003 (7)
C2	0.0268 (7)	0.0248 (7)	0.0293 (7)	0.0002 (6)	0.0002 (6)	0.0013 (6)
C3	0.0244 (7)	0.0245 (7)	0.0333 (8)	0.0023 (6)	−0.0005 (6)	−0.0009 (6)
C4	0.0276 (8)	0.0346 (9)	0.0414 (9)	−0.0006 (7)	−0.0030 (7)	−0.0028 (7)
C5	0.0289 (8)	0.0250 (7)	0.0299 (8)	−0.0004 (6)	0.0014 (6)	0.0003 (6)
C6	0.0538 (13)	0.0432 (11)	0.0627 (13)	0.0120 (9)	−0.0033 (10)	−0.0252 (10)
C7	0.0269 (8)	0.0451 (10)	0.0551 (12)	0.0004 (7)	−0.0089 (8)	−0.0086 (9)
N1	0.0257 (7)	0.0273 (7)	0.0411 (8)	0.0027 (5)	−0.0018 (6)	−0.0044 (6)
N2	0.0307 (7)	0.0281 (7)	0.0359 (7)	−0.0026 (5)	−0.0021 (6)	−0.0055 (6)
N3	0.0316 (7)	0.0269 (7)	0.0364 (7)	0.0046 (5)	0.0013 (6)	−0.0055 (6)
N4	0.0246 (6)	0.0305 (7)	0.0366 (7)	0.0018 (5)	−0.0027 (5)	−0.0025 (6)
O1	0.0334 (7)	0.0440 (8)	0.0612 (9)	0.0146 (6)	−0.0020 (6)	−0.0063 (6)
O2	0.0335 (6)	0.0302 (6)	0.0401 (7)	0.0031 (5)	−0.0052 (5)	−0.0086 (5)
O3	0.0556 (9)	0.0577 (9)	0.0556 (9)	0.0250 (7)	−0.0129 (7)	−0.0217 (7)
Cl1	0.0508 (3)	0.0742 (4)	0.0829 (4)	−0.0221 (3)	−0.0032 (3)	−0.0209 (3)
Cl2	0.0543 (3)	0.0337 (2)	0.0405 (2)	−0.00102 (19)	−0.0130 (2)	−0.00533 (17)
Cl3	0.0538 (3)	0.0503 (3)	0.0467 (3)	0.0195 (2)	−0.0059 (2)	0.0006 (2)
Zn1	0.03676 (14)	0.03346 (14)	0.04306 (15)	0.00315 (8)	−0.00657 (9)	−0.00676 (8)

### Geometric parameters (Å, º)

**Table:**

C1—O1	1.219 (2)	C6—H6B	0.9600
C1—N3	1.379 (2)	C6—H6C	0.9600
C1—N4	1.394 (2)	C7—N4	1.474 (2)
C2—O2	1.223 (2)	C7—H7A	0.9600
C2—N4	1.391 (2)	C7—H7B	0.9600
C2—C3	1.427 (2)	C7—H7C	0.9600
C3—C5	1.360 (2)	N1—H1	0.8600
C3—N1	1.380 (2)	N2—H2	0.8600
C4—N1	1.315 (2)	O3—H3A	0.7665
C4—N2	1.343 (2)	O3—H3B	0.8227
C4—H4	0.9300	Zn1—O3	2.0240 (15)
C5—N3	1.358 (2)	Zn1—Cl1	2.2121 (7)
C5—N2	1.362 (2)	Zn1—Cl2	2.2745 (6)
C6—N3	1.466 (2)	Zn1—Cl3	2.2487 (6)
C6—H6A	0.9600		
			
O1—C1—N3	121.36 (16)	N4—C7—H7C	109.5
O1—C1—N4	121.10 (16)	H7A—C7—H7C	109.5
N3—C1—N4	117.55 (14)	H7B—C7—H7C	109.5
O2—C2—N4	121.61 (15)	C4—N1—C3	108.30 (14)
O2—C2—C3	126.47 (15)	C4—N1—H1	125.8
N4—C2—C3	111.92 (14)	C3—N1—H1	125.8
C5—C3—N1	106.73 (14)	C4—N2—C5	107.74 (14)
C5—C3—C2	121.68 (15)	C4—N2—H2	126.1
N1—C3—C2	131.54 (15)	C5—N2—H2	126.1
N1—C4—N2	109.50 (15)	C5—N3—C1	117.99 (14)
N1—C4—H4	125.2	C5—N3—C6	121.95 (15)
N2—C4—H4	125.2	C1—N3—C6	119.84 (15)
N3—C5—C3	123.88 (15)	C2—N4—C1	126.70 (14)
N3—C5—N2	128.42 (15)	C2—N4—C7	117.30 (14)
C3—C5—N2	107.71 (14)	C1—N4—C7	115.90 (14)
N3—C6—H6A	109.5	Zn1—O3—H3A	119.6
N3—C6—H6B	109.5	Zn1—O3—H3B	120.0
H6A—C6—H6B	109.5	H3A—O3—H3B	105.5
N3—C6—H6C	109.5	O3—Zn1—Cl3	101.36 (5)
H6A—C6—H6C	109.5	O3—Zn1—Cl2	106.16 (6)
H6B—C6—H6C	109.5	O3—Zn1—Cl1	108.19 (5)
N4—C7—H7A	109.5	Cl1—Zn1—Cl2	111.45 (3)
N4—C7—H7B	109.5	Cl3—Zn1—Cl2	111.58 (2)
H7A—C7—H7B	109.5	Cl1—Zn1—Cl3	116.99 (3)
			
O2—C2—C3—C5	−176.95 (16)	N2—C5—N3—C1	178.75 (17)
N4—C2—C3—C5	2.2 (2)	C3—C5—N3—C6	−175.40 (18)
O2—C2—C3—N1	0.2 (3)	N2—C5—N3—C6	4.0 (3)
N4—C2—C3—N1	179.38 (17)	O1—C1—N3—C5	−175.01 (17)
N1—C3—C5—N3	179.06 (15)	N4—C1—N3—C5	5.0 (2)
C2—C3—C5—N3	−3.2 (3)	O1—C1—N3—C6	−0.2 (3)
N1—C3—C5—N2	−0.48 (18)	N4—C1—N3—C6	179.78 (18)
C2—C3—C5—N2	177.31 (15)	O2—C2—N4—C1	−178.35 (16)
N2—C4—N1—C3	0.8 (2)	C3—C2—N4—C1	2.4 (2)
C5—C3—N1—C4	−0.18 (19)	O2—C2—N4—C7	−2.2 (2)
C2—C3—N1—C4	−177.67 (18)	C3—C2—N4—C7	178.56 (15)
N1—C4—N2—C5	−1.1 (2)	O1—C1—N4—C2	173.82 (17)
N3—C5—N2—C4	−178.56 (16)	N3—C1—N4—C2	−6.1 (3)
C3—C5—N2—C4	0.95 (19)	O1—C1—N4—C7	−2.3 (3)
C3—C5—N3—C1	−0.7 (2)	N3—C1—N4—C7	177.68 (16)

### Hydrogen-bond geometry (Å, º)

**Table:**

*D*—H···*A*	*D*—H	H···*A*	*D*···*A*	*D*—H···*A*
N1—H1···O2^i^	0.86	1.87	2.7067 (18)	163
N2—H2···Cl2^ii^	0.86	2.21	3.0652 (15)	174
C6—H6*C*···O2^iii^	0.96	2.65	3.431 (3)	139
O3—H3*A*···Cl3^iv^	0.77	2.43	3.1915 (17)	176
O3—H3*B*···O1	0.82	1.90	2.718 (2)	173

Symmetry codes: (i) −*x*, −*y*+2, −*z*+1; (ii) *x*−1, *y*, *z*; (iii) *x*, −*y*+3/2, *z*−1/2; (iv) −*x*+2, −*y*+1, −*z*+1.
